# Clinical factors associated with subsequent surgical intervention in women undergoing early medical termination of viable or non-viable pregnancies

**DOI:** 10.3389/fmed.2024.1188629

**Published:** 2024-04-26

**Authors:** Heng-Kien Au, Chi-Feng Liu, Li-Wei Chien

**Affiliations:** ^1^Department of Obstetrics and Gynecology, School of Medicine, College of Medicine, Taipei Medical University, Taipei City, Taiwan; ^2^Department of Obstetrics and Gynecology, Taipei Medical University Hospital, Taipei City, Taiwan; ^3^School of Nursing, National Taipei University of Nursing and Health Science, Taipei City, Taiwan

**Keywords:** early pregnancy loss (EPL), medical termination of pregnancy, mifepristone, misoprostol, non-viable pregnancy

## Abstract

**Introduction:**

Mifepristone-misoprostol treatment for medical abortion and miscarriage are safe and effective. This study aimed to assess clinical factors associated with subsequent surgical intervention after medical termination of early viable or non-viable pregnancy.

**Methods:**

This retrospective, single-center study included women who underwent medical abortion at Taipei Medical University between January 2010 and December 2019. A total of 1,561 subjects, with 1,080 viable and 481 non-viable pregnancies, who were treated with oral mifepristone 600 mg followed by misoprostol 600 mg 48 h later were included. Data of all pregnancies and medical termination of pregnancy were evaluated using regression analysis. The main outcome was successful termination of pregnancy.

**Results:**

The success rate of medical abortion was comparable in women with viable and non-viable (92.13% vs. 92.93%) pregnancies. Besides retained tissue, more existing pregnancies with ultrasonographic findings were found in the non-viable pregnancy group than in the viable pregnancy group (29.4% vs. 14.1%, *p* = 0.011). Multivariate analysis showed that previous delivery was an independent risk factor for failed medical abortion among all included cases. In women with viable pregnancy, longer gestational age [adjusted odds ratio (aOR): 1.483, 95% confidence interval (CI): 1.224–1.797, *p* < 0.001] and previous Cesarean delivery (aOR: 2.177, 95% CI: 1.167–40.62, *p* = 0.014) were independent risk factors for failed medical abortion. Number of Cesarean deliveries (aOR: 1.448, 95% CI: 1.029–2.039, *p* = 0.034) was an independent risk factor for failed medication abortion in women with non-viable pregnancies.

**Conclusion:**

This is the first cohort study to identify risk factors for subsequent surgical intervention in women with viable or non-viable pregnancies who had undergone early medically induced abortions. The success rate of medical abortion is comparable in women with viable and non-viable pregnancies. Previous delivery is an independent risk factor for failed medical abortion. Clinical follow-up may be necessary for women who are at risk of subsequent surgical intervention.

## Introduction

Mifepristone-misoprostol has been shown to be a safe, non-invasive, and effective option for medical abortion of early pregnancy (1, 2). Studies have reported that 2.5–13% of subjects who underwent medical abortion needed subsequent surgical intervention within 8 weeks after medication administration, and the rate increased with increasing gestational weeks (3–5). Gestational age after 9 weeks, advanced maternal age, multiparity, a history of induced abortion, and previous Cesarean delivery were previously reported as independent factors affecting the outcomes (3–11). However, other reports did not find that these risk factors predicted the outcomes of medical abortion. Differences in the dosing regimens and routes of medication administration may account for the discrepant results of these studies (12, 13).

Mifepristone-misoprostol has been recommended in recent years for treating women with non-viable pregnancy. Schreiber et al. (14) reported a higher rate of complete expulsion (83.8% vs. 67.1%) and lower rate of uterine aspiration use (8.8% vs. 23.5%) in women with non-viable pregnancies in the mifepristone-misoprostol treatment group than in the misoprostol-alone group. In a systematic review and meta-analysis of randomized trials, Al Wattar et al. (15) reported that the use of mifepristone plus misoprostol was significantly more effective than misoprostol alone, with a relative risk of 1.49 (95% CI: 1.09–2.03). However, assessment of clinical factors associated with outcomes between women with early pregnancy loss and those with early medical abortion is lacking (16). The aim of the present study was to assess risk factors for subsequent surgical intervention in women with viable or non-viable early pregnancy who underwent medical abortions using the same mifepristone-misoprostol protocol.

## Materials and methods

This retrospective study was conducted in a single university-affiliated tertiary medical center. Subjects who underwent medical abortion at Taipei Medical University Hospital between January 2010 and December 2019 were enrolled. Inclusion criteria were women with maternal age over 18 with exclusion of those with medical contraindications. Subjects with missing follow-up data were also excluded. Subjects’ clinical records were examined, and data were collected from a dedicated merged database.

### Ethical considerations

The study protocol was approved by the Joint Institutional Review Board of Taipei Medical University (N202211011).

### Procedure

The protocol for medical abortion includes two mandatory visits. Before the first visit, all women have had an ultrasound scan to confirm intrauterine pregnancy and to establish gestational age. They were then treated with mifepristone (600 mg orally, Apano; Lotus, Nantou, Taiwan) followed 48 h later with misoprostol (600 mcg orally, Cytotec; Pfizer, Northumberland, United Kingdom). Fourteen to 21 days after administration of mifepristone, the women were told to return to the clinic for an interview, bimanual pelvic examination, and transvaginal ultrasound examination. Criteria for the diagnosis of non-viable pregnancy were as follows: (a) the absence of an embryo after a scan showing a gestational sac without a yolk sac; (b) the absence of an embryo after a scan showing a gestational sac with a yolk sac; (c) a non-viable embryonic pregnancy confirmed on the first scan in which the embryonic pole length was ≥7 mm; or if the embryonic pole length was <7 mm on the first scan and non-viability was confirmed based on reproducible evidence of the absence of fetal heart activity (13–15). Women with non-viable pregnancy were eligible for inclusion only with pregnancy up to 13 weeks of gestation on transvaginal sonography (TVS).

### Outcome measures

The primary outcome of the study was successful medical abortion. Success of medical abortion was defined as the non-surgical evacuation of the products of conception (POC), including: (a) complete abortion without retained POC and (b) incomplete abortion with different amounts of retained POC that were expulsed with the additional doses of misoprostol, as previously described (8). Failure was defined as the conversion of medical to surgical abortion in cases of incomplete abortion. Surgical intervention was indicated because of failure of medically induced abortion, excessive bleeding, persistent retained POC 5 weeks later, or other serious medical conditions. Existing pregnancy was defined as persistent appearance of ultrasound findings after medication. Retained tissue was defined as thickened endometrial echo-complexes in the uterine cavity.

### Statistical analysis

The discrete variables are reported as counts and percentages, and the differences between groups were tested with the Chi-square test or Fisher’s exact test as the result is relevant only when no more than 20% of cells with expected frequencies <5 and no cell have expected frequency <1 (17). Maternal age and days to surgical intervention after initial medication are presented as median and interquartile range (IQR) due to skewed distributions. Differences between two groups (successful vs. failed medical abortions or viable vs. non-viable pregnancies) for maternal age and days to surgical intervention were tested using the non-parametric Mann-Whiney U test. Analyses of the influence factors of failed medical abortion are presented by crude and adjusted odds ratios (ORs) with 95% confidence intervals (CI). Each independent variable was included in a univariable logistic regression model, from which the independent variables (except for count of parity) with *p*-values < 0.2 were stepwise included into the multivariable logistic regression model using the backward elimination method. The variable count of parity was directly excluded from multivariable analyses due to collinearity with numbers of vaginal and Cesarean deliveries. All statistical tests were two-sided with a significance level of 0.05. All statistical analyses were performed using IBM SPSS Statistics software, version 25.0 (IBM Corporation, Armonk, New York, USA).

## Results

### Subjects’ characteristics

A total of 1561 subjects treated with medical abortion were retrospectively enrolled in this study, including 1080 viable and 481 non-viable pregnancies. The diagnoses for the 481 non-viable pregnancies included 253 embryonic or fetal death, 132 yolk sac demise, and 96 anembryonic gestation. The subjects had a median maternal age of 34 years (IQR of 29–38 years) and a median gestational age of 6.6 weeks (IQR of 5.7–8.3 weeks). The clinical results included 1442 successful and 119 failed medical abortions. For the 119 subjects with failed medical abortions who needed surgery, the major indication for surgical intervention was retained tissue (*n* = 82), followed by existing pregnancy (*n* = 22) and marked vaginal bleeding or signs of infection (*n* = 15). The median period from initial medication to surgical intervention was 21 days (IQR of 16–31 days).

### Pregnancy characteristics of successful medical abortion versus failed medical abortion

The associations between pregnancy characteristics and the clinical results of medical abortion are shown in Table 1. Types of previous delivery, parity, and number of Cesarean deliveries were significantly associated with failed medical abortion. Over half of the subjects in the successful abortion group were nulliparous (56.6%) but only 42.0% were nulliparous in the failed group (*p* = 0.006). The subjects with failed medical abortions had significantly higher counts of parity than those with successful results: 58.8% of the subjects with failed results but only 43.3% of the subjects with successful results had a parity count of 1 or more (*p* = 0.011). Also, subjects with failed medical abortion had significantly more previous Cesarean deliveries than those with successful results (19.3% vs. 11.9% subjects had 1 or more Cesarean deliveries, *p* = 0.050). No significant associations were observed between medical abortion results and maternal age, gestational age, previous induced abortion, number of vaginal deliveries, or non-viable pregnancy.

**TABLE 1:** Baseline demographic and clinical characteristics of successful and failed abortion groups.

		Clinical results of medical abortion		*P*-value
		Success (*n* = 1442)	Failed (*n* = 119)	
Maternal age (years)		34.0 (28.0, 39.0)	35.0 (30.0, 38.0)	0.246
Gestational age (weeks)^†^	<9th	1,213 (84.2%)	100 (84.0%)	0.880
	9th - 11th	181 (12.6%)	16 (13.4%)	
	>11th	47 (3.3%)	3 (2.5%)	
Previous induced abortion	0	833 (57.8%)	58 (48.7%)	0.200
	1	375 (26.0%)	41 (34.5%)	
	2	149 (10.3%)	12 (10.1%)	
	3 or more	85 (5.9%)	8 (6.7%)	
Type of previous delivery	Nullipara	816 (56.6%)	50 (42.0%)	0.006*
	Vaginal	454 (31.5%)	47 (39.5%)	
	Cesarean	172 (11.9%)	22 (18.5%)	
Parity	0	817 (56.7%)	49 (41.2%)	0.011*
	1	291 (20.2%)	32 (26.9%)	
	2	279 (19.3%)	33 (27.7%)	
	3 or more	55 (3.8%)	5 (4.2%)	
Number of vaginal deliveries	0	967 (67.1%)	71 (59.7%)	0.431
	1	224 (15.5%)	22 (18.5%)	
	2	210 (14.6%)	22 (18.5%)	
	3 or more	41 (2.8%)	4 (3.4%)	
Number of Cesarean deliveries	0	1,270 (88.1%)	96 (80.7%)	0.050*
	1	108 (7.5%)	13 (10.9%)	
	2 or more	64 (4.4%)	10 (8.4%)	
Non-viable pregnancy	Yes	447 (31.0%)	34 (28.6%)	0.582
	No	995 (69.0%)	85 (71.4%)	

Maternal age is presented as median (IQR) and using the non-parametric Mann-Whitney U test. Categorical variables are presented as count (percentage) and were tested using Chi-square test. *P-value < 0.05 indicates a significant association with clinical results of medical abortion.

^†^The successful medical abortion group had one missing value for gestational age.

### Differences between viable and non-viable pregnancies

Significant differences were observed between viable and non-viable pregnancies in maternal age, gestational age, previous delivery type, count of parity, and numbers of vaginal and Cesarean deliveries. The 481 subjects with non-viable pregnancies were older (median age: 37.0 years vs. 32.0 years for viable pregnancies, *p* < 0.001). Almost all subjects with viable pregnancies had gestational age within 9 weeks (98.8%) but only 51.1% subjects with non-viable pregnancies had gestational age within 9 weeks (*p* < 0.001). Nearly two-thirds (64.7%) of the subjects with non-viable pregnancies were nulliparous but only 51.4% of the subjects with viable pregnancies were nulliparous (*p* < 0.001). Compared to the 1080 subjects with viable pregnancies, significantly fewer counts of previous parity, vaginal delivery, and Cesarean deliveries were observed in those with non-viable pregnancies (8.1% vs. 30.8% with parity of 2 or more, *p* < 0.001; 5.2% vs. 23.4% with 2 or more vaginal deliveries, *p* < 0.001; 1.9% vs. 6.0% with 2 or more Cesarean deliveries, *p* = 0.001) (Table 2).

**TABLE 2:** Baseline demographic and clinical characteristics of non-viable and viable pregnancies.

		Classification		*P*-value
		Non-viable pregnancies (*n* = 481)	Viable pregnancies (*n* = 1080)	
Maternal age (years)		37.0 (33.0, 40.0)	32.0 (27.0, 37.0)	<0.001*
Gestational age (weeks)	<9th	246(51.1%)	1,067 (98.8%)	<0.001*
	9th–11th	187 (38.9%)	11 (1.0%)	
	>11th	48 (10.0%)	2 (0.2%)	
Previous induced abortion	0	290 (60.3%)	601 (55.6%)	0.381
	1	118 (24.5%)	298 (27.6%)	
	2	45 (9.4%)	116 (10.7%)	
	3 or more	28 (5.8%)	65 (6.0%)	
Type of previous delivery	Nullipara	311 (64.7%)	555 (51.4%)	<0.001*
	Vaginal	116 (24.1%)	385 (35.6%)	
	Cesarean	54 (11.2%)	140 (13.0%)	
Parity	0	315 (65.5%)	551 (51.0%)	<0.001*
	1	127 (26.4%)	196 (18.1%)	
	2	35 (7.3%)	277 (25.6%)	
	3 or more	4 (0.8%)	56 (5.2%)	
Number of vaginal deliveries	0	365 (75.9%)	673 (62.3%)	<0.001*
	1	91 (18.9%)	155 (14.4%)	
	2	23 (4.8%)	209 (19.4%)	
	3 or more	2 (0.4%)	43 (4.0%)	
Number of Cesarean deliveries	0	426 (88.6%)	940 (87.0%)	0.001*
	1	46 (9.6%)	75 (6.9%)	
	2 or more	9 (1.9%)	65 (6.0%)	

Maternal age is presented as median (IQR) and using the non-parametric Mann-Whitney U test. Categorical variables are presented as count (percentage) and were tested using Chi-square test. *P-value < 0.05 indicates a significant association with non-viable pregnancy.

### Clinical events after failed medical abortion

For the 119 subjects with failed medical abortions, 85 were viable and 34 were non-viable pregnancies. Significant differences were found in the indications for surgical intervention between those with viable and non-viable pregnancies (*p* = 0.011); retained tissue was the major indication for surgical intervention in both groups (68.2 and 70.6%, respectively). In the non-viable pregnancy group, the other main indication was existing pregnancy (29.4%). In the viable pregnancy group, indications included ongoing pregnancy (14.1%) and marked vaginal bleeding or signs of infection (17.6%). No significant differences were found in pathology and the period from initial medication to surgical intervention between viable and non-viable pregnancies (Table 3).

**TABLE 3:** Clinical events of viable and non-viable pregnancies in women undergoing surgical intervention after failed medical abortion.

		Failed medical abortion (*n* = 119)		*P*-value
		Non-viable pregnancy (*n* = 34)	Viable pregnancy (*n* = 85)	
Indication for surgical intervention	Existing/ongoing pregnancy^a^	10 (29.4%)	12 (14.1%)	0.011*
	Marked vaginal bleeding or signs of infection	0 (0.0%)	15 (17.6%)	
	Retained tissue	24 (70.6%)	58 (68.2%)	
Pathology	No gestational tissue	3 (8.8%)	10 (11.8%)	0.756^b^
	Gestational tissue	31 (91.2%)	75 (88.2%)	
Days to surgical intervention after initial medication	≤28	20 (58.8%)	63 (74.1%)	0.101
	>28	14 (41.2%)	22 (25.9%)	
Days to surgical intervention after initial medication		26.0 (15.0, 32.0)	20.0 (16.0, 29.0)	0.316

^a^Existing pregnancy is for non-viable pregnancy, while ongoing pregnancy for viable pregnancy. Surgical intervention after initial medication is presented as median (IQR) and tested using the Mann-Whitney U test. Categorical variables are presented as count and percentage and tested using Chi-square test or ^b^Fisher’s exact test as appropriate. *P-value < 0.05 indicates a significant association between indication for surgical intervention and non-viable pregnancy.

### Pregnancy characteristics of failed medical abortion in viable pregnancies

For the 1080 viable pregnancies, gestational age, type of previous delivery, count of parity, and number of Cesarean deliveries were significantly associated with failed medical abortion. Nearly all (99.1%) viable pregnancies in the successful abortion group but only 95.3% viable pregnancies in the failed abortion group had gestational ages lower than the 9th week (*p* < 0.001). For types of previous delivery, the successful abortion group had a higher proportion of nulliparous deliveries and lower proportion of previous Cesarean deliveries compared to the failed group (nulliparous deliveries: 52.5% vs. 38.8%; Cesarean deliveries: 12.4% vs. 20.0%, *p* = 0.028). No significant associations with failed medical abortion were observed in the other pregnancy characteristics (maternal age, previous induced abortion, parity, and number of vaginal and Cesarean deliveries) (Supplementary Table 1).

### Pregnancy characteristics of failed medical abortion in non-viable pregnancies

For the 481 non-viable pregnancies, a higher count of parity (*p* = 0.019) was significantly associated with failed medical abortion. Among women who had successful medical abortions, 66.7% were nulliparous, while only half of women who had failed medical abortion were nulliparous. No significant associations with failed medical abortion were observed in the other pregnancy characteristics (maternal age, gestational age, previous induced abortion, type of previous delivery, number of vaginal and Cesarean deliveries, and diagnosis of non-viable pregnancy). No significant differences were found in the classification of miscarriage between the successful and failed cases (Supplementary Table 2).

### Risk factors of failed medical abortion

Three populations were analyzed to investigate the risk factors of failed medical abortion: all 1561 pregnancies, the 1080 viable pregnancies, and the 481 non-viable pregnancies. As mentioned in the statistical analyses section: except for count of parity, the variables with *p*-value < 0.2 in univariable analyses were stepwise included into the final multivariable models of the three populations.

For all 1,561 pregnancies, type of previous delivery, count of parity, and number of Cesarean deliveries were significantly associated with failed medication abortion in univariable analyses (Table 4). The final multivariable model included the two variables: type of previous delivery and previous induced abortion (Table 5). Also, only the type of previous delivery was significantly associated with failed medical abortion. After adjusting for previous induced abortion, women with previous vaginal delivery or previous Cesarean delivery were more likely to have failed medical abortion than those with nulliparous pregnancy, with odds ratios 1.606 (95% CI: 1.054–2.447, *p* = 0.027) and 1.994 (95% CI: 1.171–3.395, *p* = 0.011), respectively.

**TABLE 4:** Univariable analyses of the risk factors of failed medical abortion for all subjects, viable and non-viable pregnancies.

	All subjects		Viable pregnancy		Non-viable pregnancy	
	OR (95% CI)	*P*-value	OR (95% CI)	*P*-value	OR (95% CI)	*P*-value
Maternal age (years)	1.019 (0.990, 1.049)	0.204	1.028 (0.994, 1.062)	0.104	1.003 (0.934, 1.076)	0.942
Gestational age (weeks)	1.049 (0.951, 1.158)	0.338	1.476 (1.220, 1.786)	<0.001*	0.866 (0.686, 1.092)	0.223
**Type of previous delivery**						
Nullipara	reference		reference		reference	
Vaginal	1.690 (1.116, 2.557)	0.013*	1.582 (0.965, 2.594)	0.069	1.995 (0.922, 4.319)	0.079
Cesarean	2.087 (1.231, 3.538)	0.006*	2.186 (1.179, 4.053)	0.013*	1.765 (0.623, 5.003)	0.285
Previous induced abortion	1.439 (0.989, 2.092)	0.057	1.454 (0.933, 2.267)	0.098	1.382 (0.686, 2.781)	0.365
Parity	1.302 (1.082, 1.567)	0.005*	1.222 (0.988, 1.511)	0.065	1.689 (1.123, 2.540)	0.012*
Number of vaginal deliveries	1.169 (0.957, 1.427)	0.125	1.091 (0.870, 1.368)	0.452	1.563 (0.995, 2.456)	0.053
Number of Cesarean deliveries	1.455 (1.078, 1.964)	0.014*	1.393 (0.997, 1.948)	0.052	1.730 (0.864, 3.465)	0.122
Non-viable pregnancy	0.890 (0.589, 1.346)	0.582				
**Diagnosis of non-viable pregnancy**						
Anembryonic gestation	–		–		reference	
Yolk sac demise	–		–		0.902 (0.342, 2.377)	0.834
Embryonic or fetal death	–		–		0.743 (0.307, 1.796)	0.509

*P-value < 0.05 indicates a significant association between corresponding variable and failed medical abortion.

**TABLE 5:** Multivariable analyses of risk factors of failed medical abortion for all subjects, viable and non-viable pregnancies.

	All subjects		Viable pregnancy		Non-viable pregnancy	
	OR (95% CI)	*P*-value	OR (95% CI)	*P*-value	OR (95% CI)	*P*-value
Gestational age (weeks)	–		1.483 (1.224, 1.797)	<0.001*	–	
Type of previous delivery	–		–		–	
Nullipara	Reference		Reference		–	
Vaginal	1.606 (1.054, 2.447)	0.027*	1.630 (0.990, 2.686)	0.055	–	
Cesarean	1.994 (1.171, 3.395)	0.011*	2.177 (1.167, 4.062)	0.014*	–	
Previous induced abortion	1.304 (0.891, 1.911)	0.172	–		–	
Number of vaginal deliveries	–		–		1.140 (0.904, 1.438)	0.267
Number of Cesarean deliveries	–		–		1.448 (1.029, 2.039)	0.034*

*P-value < 0.05 indicates a significant association between corresponding variable and failed medical abortion.

Univariable analysis revealed that viable pregnancies with longer gestational age and previous Cesarean deliveries were significantly associated with failed medical abortion, and the likelihood of failed medical abortion was increased with every week of gestational age, with odds ratio of 1.476 (*p* < 0.001). Subjects who had previous Cesarean deliveries were more likely to have failed medical abortions than those who were nulliparous, with the odds ratio of 2.186 (*p* = 0.013) (Table 4). In the final multivariable model, the likelihood of failed medical abortion was increased with increasing gestational age, with the adjusted odds ratio of 1.483 (95% CI: 1.224–1.797, *p* < 0.001); after adjusting for gestational age, subjects with previous Cesarean delivery were more likely to have failed medical abortion than those who were nulliparous, with the odds ratio of 2.177 (95% CI: 1.167–4.062, *p* = 0.014) (Table 5).

In univariable analysis, for non-viable pregnancies, only counts of parity were significantly associated with failed medical abortion (*p* = 0.012) (Table 4). The two variables, number of vaginal deliveries and Cesarean deliveries, were included in the final multivariable model. Only the number of Cesarean deliveries was significantly associated with failed medical abortion. The likelihood of failed medical abortion increased with increased counts of Cesarean deliveries, with the adjusted odds ratio of 1.448 (95% CI: 1.029–2.039, *p* = 0.034) (Table 5).

## Discussion

To the best of our knowledge, this is the first cohort study to explore risk factors for subsequent surgical intervention in women with viable or non-viable pregnancies who underwent medical abortion in a single center using the same medical regimen.

### Principal findings

Gestational age and previous Cesarean delivery are associated with subsequent surgical intervention in women with viable pregnancies. Only the number of previous Cesarean deliveries was associated with failed medical abortion in women with non-viable pregnancies, suggesting that clinical follow-up after medical abortion may be necessary for women at risk of subsequent surgical intervention. Gestational age was associated with the need for subsequent surgical intervention in the viable pregnancy group but not in the non-viable pregnancy.

### Results in the context of what is known

Previous studies have shown an increased risk of surgical intervention with increasing gestational age up to week 9 in viable pregnancies (4, 7). No significant differences were found in the success rate among the gestational age groups of <9 weeks, 9–11 weeks and >11 weeks in non-viable pregnancies. It is interesting to note that women with mifepristone-pretreatment had a lower success rate than those receiving misoprostol alone in the gestational age group of 10 to 12 weeks in a previous randomized trial (14). Results of the present study confirmed that gestational age is not a concern for women with early pregnancy loss who undergo medical abortion.

Nulliparity was shown to be a strong predictor for the success of medical abortions in early pregnancy (4, 7). The present study also showed higher odds for failed medical abortion in subjects with previous delivery regardless of delivery type. Prefumo et al. (18) showed that endovascular trophoblast invasion in early pregnancy is more extensive in parous women than in nulliparous women, suggesting that higher adherence of decidual vessels in parous women may be partly responsible for this outcome. The underlying mechanisms, however, remain to be determined.

Previous studies have reported an association between increased odds of surgical intervention after medical abortion and prior Cesarean delivery (8, 10). Cesarean section may injury uterine muscles, reduce physiologic contractions, and consequently impede the expulsion of gestational tissues during the process of medical abortion. This is illustrated in studies showing that a history of Cesarean delivery is associated with uterine rupture in women who underwent medical abortions at gestational 15–35 weeks (19, 20). Intrauterine scars due to Cesarean delivery may contribute to the outcome of failed medical abortion. Ofili-Yebovi et al. (21) reported lower-segment uterine scars detected by sonography in 99% of women with a history of Cesarean section. Our previous study analyzing 183 women between 6 and 8 gestational weeks who underwent medical abortion reported a significantly higher failure rate in subjects with uterine scar defects due to previous Cesarean section than in those without (22). In the present study, the association between failed medical abortion and number of Cesarean deliveries in the non-viable pregnancy group also illustrates that repeated injuries tend to hinder uterine muscle contraction.

### Clinical implications

The success rate of medical abortion varies between studies because of differences in treatment protocols and doses (3–7, 10, 11). The overall rate of successful management of early pregnancy termination with mifepristone (600 mg) followed by misoprostol (600 mg) was 92.4% in the present study, which was similar between viable and non-viable pregnancy groups. The mifepristone dosage of 600 mg is higher than the WHO-recommended 200 mg for medical abortions. We followed the guidelines of the local administration and manufacturers’ recommendations regarding mifepristone dosage. Regarding misoprostol, many different treatment regimens are being used, since the optimal regimen regarding dosage and route of administration for misoprostol remains unclear to date. We used the oral administration of 600 mg misoprostol, which is comparable to that used in the Triple M trial by Hamel et al. (23) which used oral administration of 800 mg misoprostol. Meanwhile, Schreiber et al. (14) conducted a randomized controlled trial, the Pregnancy Failure Management Regimens trial, assessing the use of mifepristone in the setting of early pregnancy loss. In the mifepristone and misoprostol groups of that study, 83.8% of patients passed the gestational sac with no additional intervention, which is consistent with our results (92.9%, 447 out of 481). Differences in the odds for surgical intervention were not statistically significant between the different clinical diagnoses of non-viable pregnancy. In addition, the non-viable pregnancy group had a significantly higher percentage of subjects with existing pregnancy than the viable pregnancy group, and none reported vaginal bleeding or signs of infection. Blavier et al. (24), who analyzed the clinical outcomes of 104 women with non-viable pregnancies, found that the serum progesterone level at initial diagnosis predicted the probability of surgical evacuation and the delay before non-surgical evacuation. Thus, the level of progesterone secreted by the gestational tissue may play a role in determining the effects of mifepristone in these women. However, further investigation may be needed to elucidate the possible mechanism.

### Research implications

Results of the present study suggest that further prospective controlled studies with a larger sample from multiple centers are needed to elucidate the optimal route of misoprostol and patient satisfaction.

### Strengths and limitations

The strengths of the present study are the lack of selection bias due to the inclusion of all early medical abortions for both viable and non-viable pregnancies as well as their complete clinical follow-up in a single center. Furthermore, we analyzed the clinical impact factors by using univariable and multivariable regression analysis. Previous deliveries, especially Cesarean delivery, may have an impact on the outcomes of treatment. We noted that treatment success with mifepristone pretreatment followed by misoprostol in early pregnancy loss was not inferior as in viable pregnancy, and the results of this analysis further support that no baseline clinical factors should restrict women for medical termination of early pregnancy. Therefore, the identification of clinical confounding factors may help in determining follow-up protocols. The study also has several limitations. Interpretation of study results is limited by the retrospective nature of the study, which may limit generalization of results to other populations, and does not rule out selection bias, which may limit patient follow-up and inferences of causality. The quality of our measurements may be influenced by changes in documentation over the tenure of the project such as implementation of an electronic medical record. Also, this study was not controlled in the same gestational period, though we determined differences between viable and non-viable pregnancies.

## Conclusion

The success rate of medical abortion is comparable in women with viable and non-viable pregnancies. Failure of early medical abortion is associated with gestational age and previous Cesarean delivery in women with viable pregnancy, while it is only associated with the number of Cesarean deliveries in non-viable pregnancy. Results of the present study may provide useful information by which to counsel patients or to help identify women at risk for subsequent surgical intervention, for whom clinical follow-up may be necessary.

## Data availability statement

The original contributions presented in this study are included in this article/Supplementary material, further inquiries can be directed to the corresponding author.

## Ethics statement

The study protocol was approved by the Joint Institutional Review Board of Taipei Medical University (TMU-JIRB No. N202211011). The studies were conducted in accordance with the local legislation and institutional requirements.

## Author contributions

H-KA: acquisition of data, analysis and interpretation of data, drafting of the manuscript, critical revision of the manuscript, final approval of the manuscript, statistical analysis, clinical studies, administrative, technical or material support. L-WC: conception and design, acquisition of data, analysis and interpretation of data, drafting of the manuscript, final approval of the manuscript, guarantor of integrity of the entire study, definition of intellectual content, literature research, clinical studies, supervision. All authors contributed to the article and approved the submitted version.
