# Supplementary material for: Clinical factors associated with subsequent surgical intervention in women undergoing early medical termination of viable or non-viable pregnancies
Source: Front Med (Lausanne). 2024 Apr 26;11:1188629. doi: 10.3389/fmed.2024.1188629 (PMC11082305; doi:10.3389/fmed.2024.1188629)
Supplement: Supplementary file 1 [file Table_1.docx]

Supplementary Table 1. Comparison between successful and failed medical abortion by pregnancy characteristics in women with viable pregnancies.

|  | | Viable pregnancy | | P-value |
| --- | --- | --- | --- | --- |
|  |  | Successful (n=995) | Failed (n=85) |  |
| Maternal age (years) | | 32.0 (27.0, 37.0) | 34.0 (29.0, 38.0) | 0.101 |
| Gestational age (weeks) | <9th | 986 (99.1%) | 81 (95.3%) | <0.001 ^a^* |
|  | 9th - 11th | 7 (0.7%) | 4 (4.7%) |  |
|  | >11th | 2 (0.2%) | 0 (0.0%) |  |
| Previous induced abortion | 0 | 561 (56.4%) | 40 (47.1%) | 0.227 |
|  | 1 | 267 (26.8%) | 31 (36.5%) |  |
|  | 2 | 107 (10.8%) | 9 (10.6%) |  |
|  | 3 or more | 60 (6.0%) | 5 (5.9%) |  |
| Type of previous delivery | Nullipara | 522 (52.5%) | 33 (38.8%) | 0.028* |
|  | Vaginal | 350 (35.2%) | 35 (41.2%) |  |
|  | Cesarean | 123 (12.4%) | 17 (20.0%) |  |
| Parity | 0 | 519 (52.2%) | 32 (37.6%) | 0.054 |
|  | 1 | 174 (17.5%) | 22 (25.9%) |  |
|  | 2 | 250 (25.1%) | 27 (31.8%) |  |
|  | 3 or more | 52 (5.2%) | 4 (4.7%) |  |
| Number of vaginal deliveries | 0 | 624 (62.7%) | 49 (57.6%) | 0.780 |
|  | 1 | 141 (14.2%) | 14 (16.5%) |  |
|  | 2 | 190 (19.1%) | 19 (22.4%) |  |
|  | 3 or more | 40 (4.0%) | 3 (3.5%) |  |
| Number of Cesarean deliveries | 0 | 872 (87.6%) | 68 (80.0%) | 0.132 |
|  | 1 | 66 (6.6%) | 9 (10.6%) |  |
|  | 2 or more | 57 (5.7%) | 8 (9.4%) |  |

Maternal age is presented as median (IQR) and was tested using the non-parametric Mann-Whitney U test. Categorical variables are presented as count (percentage) and were tested using Chi-square test or ^a^ Fisher’s exact test, as appropriate. *P-value <0.05 indicates a significant association between corresponding variable and clinical results of medical abortion.

Supplementary Table 2. Comparison between successful versus failed medical abortion by pregnancy characteristics in women with non-viable pregnancies.

|  | | Nonviable pregnancy (N=481) | | P-value |
| --- | --- | --- | --- | --- |
|  |  | Success (n=447) | Failed (n=34) |  |
| Maternal age (year) | | 37.0 (33.0, 40.0) | 37.0 (35.0, 40.0) | 0.773 |
| Gestational age (weeks) | <9th | 227 (50.8%) | 19 (55.9%) | 0.848 |
|  | 9th - 11th | 175 (39.1%) | 12 (35.3%) |  |
|  | >11th | 45 (10.1%) | 3 (8.8%) |  |
| Previous induced abortion | 0 | 272 (60.9%) | 18 (52.9%) | 0.419^a^ |
|  | 1 | 108 (24.2%) | 10 (29.4%) |  |
|  | 2 | 42 (9.4%) | 3 (8.8%) |  |
|  | 3 or more | 25 (5.6%) | 3 (8.8%) |  |
| Type of previous delivery | Nullipara | 294 (65.8%) | 17 (50.0%) | 0.179 |
|  | Vaginal | 104 (23.3%) | 12 (35.3%) |  |
|  | Cesarean | 49 (11.0%) | 5 (14.7%) |  |
| Parity | 0 | 298 (66.7%) | 17 (50.0%) | 0.019^a^* |
|  | 1 | 117 (26.2%) | 10 (29.4%) |  |
|  | 2 | 29 (6.5%) | 6 (17.6%) |  |
|  | 3 or more | 3 (0.7%) | 1 (2.9%) |  |
| Number of vaginal deliveries | 0 | 343 (76.7%) | 22 (64.7%) | 0.079^a^ |
|  | 1 | 83 (18.6%) | 8 (23.5%) |  |
|  | 2 | 20 (4.5%) | 3 (8.8%) |  |
|  | 3 or more | 1 (0.2%) | 1 (2.9%) |  |
| Number of Cesarean deliveries | 0 | 398 (89.0%) | 28 (82.4%) | 0.183^a^ |
|  | 1 | 42 (9.4%) | 4 (11.8%) |  |
|  | 2 or more | 7 (1.6%) | 2 (5.9%) |  |
| Diagnosis of nonviable pregnancy | Anembryonic gestation | 88 (19.7%) | 8 (23.5%) | 0.779 |
|  | Yolk sac demise | 122 (27.3%) | 10 (29.4%) |  |
|  | Embryonic or fetal death | 237 (53.0%) | 16 (47.1%) |  |

Maternal age is presented as median (IQR) and was tested using the non-parametric Mann-Whitney U test. Categorical variables are presented as count (percentage) and were tested using Chi-square test or ^a^ Fisher’s exact test, as appropriate. *P-value<0.05 indicates a significant association between corresponding variable and clinical results of medical abortion.

† The successful abortion group had one missing value in gestational age.
